# PB28, the Sigma-1 and Sigma-2 Receptors Modulator With Potent Anti–SARS-CoV-2 Activity: A Review About Its Pharmacological Properties and Structure Affinity Relationships

**DOI:** 10.3389/fphar.2020.589810

**Published:** 2020-12-07

**Authors:** Carmen Abate, Mauro Niso, Francesca Serena Abatematteo, Marialessandra Contino, Nicola Antonio Colabufo, Francesco Berardi

**Affiliations:** Dipartimento di Farmacia-Scienze del Farmaco, Università degli Studi di Bari ALDO MORO, Bari, Italy

**Keywords:** sigma-1 receptor, sigma-2 receptor, SARS-CoV-2, PB28, structure-affinity relationships

## Abstract

These unprecedented times have forced the scientific community to gather to face the COVID-19 pandemic. Efforts in diverse directions have been made. A multi-university team has focused on the identification of the host (human) proteins interacting with SARS-CoV-2 viral proteins, with the aim of hampering these interactions that may cause severe COVID-19 symptoms. Sigma-1 and sigma-2 receptors surprisingly belong to the “druggable” host proteins found, with the pan-sigma receptor modulator PB28 displaying the most potent anti–SARS-CoV-2 activity in in vitro assays. Being 20-fold more active than hydroxychloroquine, without cardiac side effects, PB28 is a promising antiviral candidate worthy of further investigation. Our research group developed PB28 in 1996 and have thoroughly characterized its biological properties since then. Structure–affinity relationship (SAfiR) studies at the sigma receptor subtypes were also undertaken with PB28 as the lead compound. We herein report our knowledge of PB28 to share information that may help to gain insight into the antiviral action of this compound and sigma receptors, while providing structural hints that may speed up the translation into therapeutics of this class of ligands.

## Introduction

**FIGURE 1:**
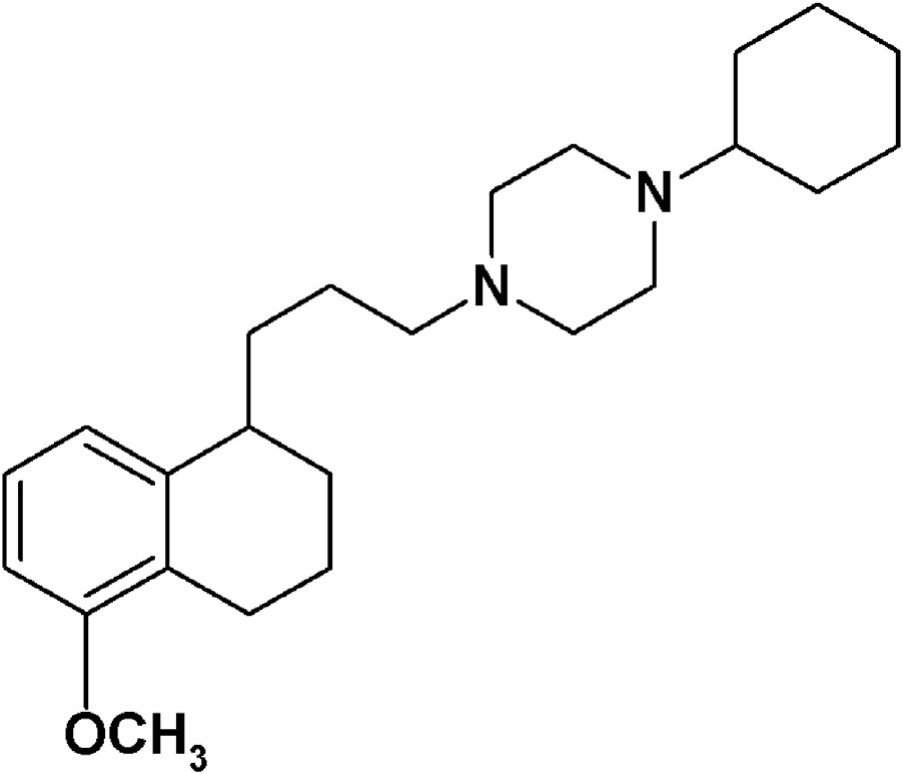
PB28.

In April 2020, a multi-university team mapped the interactions between human proteins and SARS-CoV-2, with the aim of finding existing drugs that interact with these proteins and speed up the discovery of anti-SARS-CoV-2 drugs that lessen the severity of COVID-19 symptoms (Gordon et al., 2020). Most of the viral proteins (26 out of 29) were expressed in human cells, and 332 human proteins interacting with them were identified by affinity-purification mass spectrometry. Sixty-six host proteins were ‘druggable’ by 69 drugs (FDA-approved or in development), and sigma receptors, emerged for their interaction with the NSP6 and ORf9c coronavirus proteins (Gordon et al., 2020). Sigma receptors, which are divided in two subtypes, namely sigma-1 and sigma-2, are endoplasmic binding sites with a still not very clear mechanism of action despite their first discovery in 1976 (Martin et al., 1976; Abate et al., 2018; Schmidt and Kruse, 2019). The sigma-1 subtype was cloned in the early 1990s and defined as a pluripotent chaperone (Su et al., 2016). It is mainly localized at the endoplasmic reticulum–mitochondria associated membranes (MAM) where it regulates Ca^++^ fluxes through interaction with the binding immunoglobulin protein (BiP) (Ortega-Roldan et al., 2013). In addition, regulation of K^+^ channels has been reported for this subtype (Abraham et al., 2019). Through its interaction with a number of client proteins, sigma-1 subtype is able to modulate several functions, such as neuroprotection, also through regulation of microglia activity, neuroregulation, and modulation of the proliferative status of cells (Hall et al., 2009; Moritz et al., 2015; Tsai and Su, 2017; Morales-Lázaro et al., 2019; Tesei et al., 2019). Therefore, ligands acting at the sigma-1 receptors have several therapeutic potentials such as treatment of Huntington’s (Eddings et al., 2019), Parkinson’s (Francardo et al., 2019), and Alzheimer’s diseases (Hall et al., 2018; Maurice et al., 2019); chronic pain (Merlos et al., 2017); cocaine abuse; depression; amnesia; and tumors (Chevallier et al., 2011; Maurice and Goguadze, 2017; Su, 2019). Treatment of Amyotrophic lateral sclerosis (ALS), in which association with a mutation in the sigma-1 subtype was reported, is also under study with sigma-1 receptor ligands (Al-Saif et al., 2011; Ionescu et al., 2019; Couly et al., 2020). A good number of clinical studies are in progress to determine the clinical utility of these receptors with small molecule ligands used as diagnostics (^18^F-FTC-146, ClinicalTrials.gov identifier NCT04314856) or therapeutics (pridopidine, AVP-923, ClinicalTrials.gov identifier NCT03019289 and NCT01324232). The recently obtained unique crystal structure accounts for the sigma-1 chaperone activity through polymerization–oligomerization association in which the ligand-driven conformation of the α4 helix is determinant (Alon et al., 2017b). The lesser known sigma-2 subtype has only recently been identified as the TMEM97 protein after previous controversies about its identity (Colabufo et al., 2006; Xu et al., 2011; Abate et al., 2015a; Alon et al., 2017a; Pati et al., 2017a). Ligands acting at the sigma-2 receptors have mostly antitumor actions, being the sigma-2 subtype overexpressed in tumor cells where it regulates cell death through different pathways (Wheeler et al., 2000; Hornick et al., 2012a; Hornick et al., 2012b; Zeng et al., 2012; Pati et al., 2017b). Positron emission tomography (PET) tracers for the diagnosis of diverse tumor forms have been developed, and one of these tracers is under clinical study (^18^F-ISO-1) (ClinicalTrials.gov identifier NCT00968656; NCT02204202). However, a recently developed class of sigma-2 antagonists is able to displace Aβ oligomers from sigma-2 neuronal receptors reducing the Aβ neurotoxic effects and shows promise in the treatment of Alzheimer’s disease (Izzo et al., 2014a; Izzo et al., 2014b). One of the studied sigma-2 antagonists, named Elayta™ (or CT1812), has entered a phase 2 clinical trial with 540 patients with early Alzheimer’s disease (Grundman et al., 2019) (ClinicalTrials.gov identifier NCT03522129). Besides the listed therapeutic potentials associated with sigma-1 and sigma-2 receptor modulation, the work by Gordon et al. (2020) has shed light on the novel potential of sigma receptors for the treatment of the SARS-CoV-2 infection. Among all the compounds tested, interacting with sigma receptors or with the other host targets, 1-cyclohexyl-4-[3-(5-methoxy-1,2,3,4-tetrahydronaphthalen-1-yl)propyl]piperazine (PB28; Figure 1) has emerged for its up to 20-fold more potent antiviral effect than hydroxychloroquine, with less likely cardiac side effects (Gordon et al., 2020). One of the raised hypotheses for the effect of PB28 concerns its binding to inositol trisphosphate receptor (InsP3) in the endoplasmic reticulum and a consequent interference with the autophagosome production modulated by the virus (Colabufo et al., 2020). Through a bioinformatic study, Sauvat et al. (2020) have recently suggested that the anti–SARS-CoV-2 properties of PB28 and other “repurposed” compounds are linked to their lysosomotropic action, as for chloroquine and hydroxychloroquine. Nevertheless, the many and diverse modes of action suggested for the latter compounds as antiviral agents are still under study (Rolain et al., 2007; Tripathy et al., 2020). Additionally, recent findings show how the phosphorylation state of host and virus proteins has a key role in the COVID-19 infection. Upregulation and downregulation of several kinases with dysregulation of the downstream signaling pathways were found in SARS-CoV-2 infection, widening the directions in which the possible mechanisms of action of repurposed anti- COVID drugs need to be investigated (Bouhaddou et al., 2020). PB28 is a subnanomolar affinity sigma-1 and sigma-2 receptors ligand that was produced in our laboratory in the 1990s (Berardi et al., 1996; Berardi et al., 2004; Berardi et al., 2009a). Its promising sigma receptor profile prompted PB28 biological characterization, making it a tool for studies related to sigma receptors. Additionally, PB28 became our lead compound for structure–affinity relationship (SAfiR) studies.

The aim of the present review is to provide all the information available in our hands about PB28 in order to help with the understanding of its mechanism of action against SARS-CoV-2 and to provide hints for structural modifications to speed up the development of antiviral therapeutics to face the COVID-19 pandemic.

## Biological Evaluation of PB28

### PB28 Affinity: *In vitro* Evaluation

The affinity evaluation was performed according to the sigma-1 and sigma-2 binding protocols that were adjusted through the years (Berardi et al., 1996; Berardi et al., 2009a; Berardi et al., 2009b). PB28 was tested in the election tissues for the sigma-1 (guinea pig brain) and sigma-2 (rat liver) receptors, showing excellent affinity for both (Table 1) (Berardi et al., 2009a). Moreover, the selectivity profile was deepened by testing PB28 affinity toward other systems as reported in Table 1.

**TABLE 1:** PB28 affinity values.

Receptor	Sigma-1	Sigma-2	5-HT_1A_	D_2_	α1	5-HT_3_	κ	δ	μ	ORL1	EBP
*K*_i_, nM	0.38^a^	0.68^a^	258	604	>7,500^b^	>700^b^	460	>2,500^b^	445	>2,500^b^	8.38^c^

ORL1, opioid receptor like-1; EBP, emopamil binding protein.

a Berardi et al., 2009a.

b Berardi et al., 1996.

c Berardi et al., 2009b.

### PB28 Activity: *Ex Vivo* Evaluation in Organ Bath Experiments

As for the activity evaluation, PB28 was initially tested in the isolated organ bath experiment assay, in guinea pig bladder and ileum, where both sigma receptors are present (Colabufo et al., 2003). With this assay, the activity profile of a sigma receptor ligand may be suggested, because an agonist is able to inhibit the electrically evoked contraction in both organs (Colabufo et al., 2003). To split sigma-2 from sigma-1 activity in ligands with a mixed sigma-1/2 profile, we carried out the experiments in sigma-1 desensitized and sigma-1 non-desensitized media (Colabufo et al., 2003).

As from Table 2, PB28, endowed with subnanomolar sigma-1 and sigma-2 receptor affinities, has been tested in guinea pig bladder and ileum in both setups. Comparable results were obtained in the two experimental conditions that suggest sigma-2 agonist behavior.

**TABLE 2:** Inhibition of electrically evoked contraction in guinea pig bladder and ileum in sigma-1 desensitized or non-desensitized setups.

Compound	Bladder		Ileum	
PB28	Non-desensitized	Desensitized	Non-desensitized	Desensitized
EC_50_ value (μM)	5.80^a^	2.62^a^	3.78^a^	3.96^a^

a Colabufo et al., 2003.

### PB28 Activity: *In Vitro* Evaluation in Tumor Cell Lines

Then, PB28 cytotoxicity in two tumor cell lines was investigated in order to confirm the behavior of the compound at the sigma-2 receptor and to define its sigma-1 mediated activity. Indeed, it is reported that sigma-2 agonists and sigma-1 antagonists induce cytotoxicity in tumor cell lines (Colabufo et al., 2004). Rat C6 glioma and human neuroblastoma SK-N-SH cells that physiologically express the two receptors subtypes were selected for this purpose (Colabufo et al., 2004). The choice of these two cell lines has been driven by the observation that both cell lines express the sigma-2 subtype with high density, while the sigma-1 subtype is expressed in two different affinity states: high affinity in C6 and low affinity in SK-N-SH cells (Colabufo et al., 2004). The antiproliferative (by MTT assay) and cytotoxic (by LDH assay) effects were induced in both cell lines, suggesting that PB28 is a sigma-1 antagonist and a sigma-2 agonist (Table 3). These findings proposed PB28 as a potential antitumor agent prompting its investigation in several tumor cell lines. PB28 cytotoxicity was also tested in chemoresistant tumors, in two different cell lines where sigma-2 receptors are overexpressed: the chemosensitive breast adenocarcinoma cancer cells MCF7 and the resistant doxorubicine counterpart MCF7dx cell line. The latter cell line differs from the former for its high density of glycoprotein-P (P-gp), an efflux pump considered as one of the main causes of multidrug resistance (MDR). In both MCF7 and MCF7dx cells, PB28 inhibited cell growth, inducing a cell cycle arrest in the G0–G1 phase and a caspase-independent apoptosis. These effects were all sigma-receptor mediated (Azzariti et al., 2006).

**TABLE 3:** PB28 affinity and activity profile in C6 rat glioma and SK-N-SH human neuroblastoma cell lines.

Sigma-2 *K*i, nM		Antiproliferative effect EC_50_, μM		Cytotoxicity EC_50_, μM	
SK-N-SH	C6	SK-N-SH	C6	SK-N-SH	C6
1.78^a^	0.89^a^	0.31^a^	0.45^a^	8.13^a^	15^a^

a Colabufo et al., 2004.

### PB28 and Multidrug Resistant Proteins

The downregulation of P-gp induced by PB28 observed in the chemoresistant phenotype, i.e., MCF7dx cells, was of great interest. The reduction of P-gp expression induced by PB28 was specific, cell-type independent, and not related to a generalized inhibition of protein synthesis. This observation together with the data reported in the literature that demonstrate the same effect for other sigma-2 receptor agonists (Bowen et al., 1997), prompted us to co-administer PB28 with doxorubicin. Doxorubicin, a first-line antitumor drug in breast cancer, is a P-gp substrate. Thus, it is effluxed by the pump, resulting in resistant tumors overexpressing P-gp. The combination strategy was successful since an increase of intracellular accumulation of doxorubicin was shown when the antitumor agent was co-administered with PB28. These findings encouraged further study about the interaction of PB28 with P-gp in order to rationalize the enhancement of doxorubicin efficacy in drug-resistant tumors upon co-administration with PB28. With this purpose, PB28 underwent a combination of three biological assays: 1) inhibition of the transport of a radiolabeled P-gp substrate; 2) determination of its efflux through a monolayer, represented by the permeability value *P*_app_ ratio; and 3) the effect on ATP cell depletion (Polli et al., 2001; Colabufo et al., 2008b). Briefly, a P-gp substrate is able to compete with the transport of a (radiolabeled) P-gp substrate, induces ATP consumption (being transported), and has a permeability value (P_*app*_ ratio) ≥2. A P-gp inhibitor is also able to compete with the transport of a radiolabeled P-gp substrate, but it does not induce an ATP consumption (not being transported) and has a permeability value (P_*app*_ ratio) ≤2. Results from these assays (reported in Table 4) demonstrated that PB28 is a P-gp inhibitor, and novel classes of potent P-gp modulators were obtained through structural changes on PB28 (Colabufo et al., 2008b; Colabufo et al., 2009). Since MDR is also caused by other P-gp sister proteins, such as the breast cancer resistance protein (BCRP) and multidrug resistance-associated protein 1 (MRP1), we also tested PB28 activity versus these proteins. An effect only toward BCRP, that is the P-gp monomer highly expressed in breast cancer, was observed (Table 4).

**TABLE 4:** P-gp interacting profile and selectivity toward all the MDR proteins of PB28.

P-gp transport inhibition	ATPase activation	Monolayer efflux *P*_app ratio_ (BA/AB)	MDCK-MDR1	MDCK-BCRP	MDCK-MRP1
EC_50_, μM			EC_50_, μM		
0.55^a^	NO^a^	1.7^a^	3.0^b^	20^b^	>100^b^

a Data obtained with the Vinblastine-based assay (Colabufo et al., 2008b).

b Data obtained with the Calcein-AM–based assay (Colabufo et al., 2009).

### PB28 Activity: *In Vitro* Evaluation of Signaling Pathways in Tumor Cell Lines

In the last few years, the antiproliferative effect (Table 5), caspase-3 activation, ROS, lysosomal membrane permeabilization (LMP), and mitochondrial superoxide generation induced by PB28 have been evaluated in several cell lines, such as human prostatic cancer PC3 cells, hippocampal mouse HT-22 cells, breast cancer cells (MCF7, MCF7dx, MCF7σ1), and different pancreatic cancer cells (Panc02, KP02, KCKO, MIAPaCa-2, BxPC3, AsPC-1, Panc-1) (Azzariti et al., 2006; Abate et al., 2011c; Abate et al., 2012; Hornick et al., 2012b; Niso et al., 2013a; Pati et al., 2017b). The data are summarized in Table 5. Recapitulating, a moderate cytotoxic effect was exerted in all the cell lines tested except for some pancreatic cancer cells such as Panc-1, KP02, and AsPC1 (EC50 > 100 μM). In contrast to other sigma receptor ligands, PB28 did not induce caspase-3 activation but increased ROS production in the cell lines tested (MCF7, MCF7dx, and Panc02). Additionally, LMP and mitochondrial superoxide generation were recorded upon PB28 administration to pancreatic cells, with strong increase in the cells most sensitive to PB28. These pieces of evidence together show that oxidative stress is at least in part responsible of PB28 cytotoxicity (Hornick et al., 2012b).

**TABLE 5:** PB28 antiproliferative activity and activated death pathways.

Cell line	Antiproliferative activity (EC_50_, μM)	Caspase-3 activation	ROS involvement	Superoxide generation
Prostatic				
PC3	37.7^a^	—	—	—
Hippocampal				
HT22	30.0^b^	—	—	—
Breast				
MCF7	28.4^c^	No^d^	Yes^c^	—
MCF7adr	77.5^c^	No^d^	Yes^c^	—
MCF7σ1	31.2^b^	—	—	—
Pancreatic				
Panc02	43^e^	No^e^	Yes^e^	Yes^e^
Panc-1	>100^e^	—	—	—
KP02	>100^e^	—	—	Yes^e^
KCKO	87^e^	—	—	—
MIAPaCa-2	51^e^	—	—	—
BxPC3	96^f^	—	Yes	Yes^e^
AsPC-1	>100^e^	—	Yes	Yes^e^

a Abate et al., 2011c.

b Abate et al., 2012.

c Niso et al., 2013a.

d Azzariti et al., 2006.

e Pati et al., 2017b.

f Hornick et al., 2012b.

### PB28 Activity: *In Vivo* Evaluation

Together with other sigma ligands, PB28 was also tested in preclinical models of pancreatic tumor (BxPC3 in athymic nude mice and Panc02 tumor in C57BL/6 female mice). In these experiments, PB28 significantly reduced tumor volume, to the same extent of the standard treatment (gemcitabine), and conferred a survival advantage. Importantly, no signs of toxicity (serum analyses and body weight) were observed in tumor-bearing and non-tumor-bearing mice under the same conditions, and the treatment was well tolerated (Hornick et al., 2012b; Pati et al., 2017b).

### PB28 and Calcium Homeostasis in Tumor Cell Lines

The widely reported role of sigma receptors in calcium signaling was also taken into account for the biological characterization of PB28 (Cassano et al., 2006). Sigma-2 agonists elicit two different types of intracellular calcium increases: first, a transient increase, due to calcium mobilization from the endoplasmic reticulum, followed by a second sustained increase, due to calcium mobilization from mitochondria (Vilner and Bowen, 2000). Thus, calcium mobilization was probed for PB28 in SK-N-SH cells. While no effect was found after a rapid administration, a prolonged incubation (45 min) of PB28 was able to abolish the cytosolic calcium increase due to the stimulation exerted by physiological agonists such as carbachol or histamine. This study led to the pivotal finding that PB28 is able to interfere with calcium mobilization from the endoplasmic reticulum by a direct modulation of InsP3-sensitive and caffeine-sensitive calcium release in SK-N-SH cells (Cassano et al., 2006). Given the sigma-1 subnanomolar affinity, the effect of PB28 on calcium mobilization was also probed in a sigma-1 cell model developed in our laboratory, i.e., MCF7 cells stably transfected with human sigma-1 receptor (MCF7σ1 cell line) (Abate et al., 2012). For this test, cells that express sigma receptors to different extents were chosen, with a particular focus on MCF7σ1 cells and the wild-type counterpart MCF7. PB28 was able to inhibit the increase in calcium mobilization mediated by bradykinin in all the cell lines studied, with low or high expression of both receptors. By contrast, (+)-pentazocine, that is the sigma-1 putative agonist, increased the bradykinin-mediated calcium fluxes in the cell lines where sigma-1 receptors are significantly present. Thus, the hypotheses that bradykinin-triggered calcium mobilization is sigma-mediated and that PB28 biological effects result from its opposite activity at the two sigma receptors (antagonism at sigma-1 and agonism at sigma-2 receptors) were corroborated (Cassano et al., 2006).

## PB28: Structural Insights

### PB28 and Histones

With the aim to contribute to the elucidation of the sigma-2 receptor identity, which was not yet disclosed, PB28 was used to identify its interacting proteins (Colabufo et al., 2006). An amino group was inserted in the 8-position of the tetralin nucleus in order to functionalize a NHS-ester activated sepharose column, through which a lysate of proteins from the human SK-N-SH neuroblastoma cells was eluted. That cell line was selected because of the good density of sigma-2 receptor and the presence of the sigma-1 receptor in the low affinity state, as described earlier. The SDS-page bands were excised and analyzed through MALDI-MS and LC-MS-MS spectroscopy and six eluted proteins (with molecular weight ranging from 13 to 26 kDa) were identified as histone proteins (H1, H1.2, H2A.5, H2B, H3.3a) together with the 40S ribosomal protein S3. Saturation of the eluted proteins with [^3^H]-DTG was consistent with data obtained with the same radioligand in SK-N-SH cell homogenate (*K*_d_ = 20.3 nM and B_max_ = 588 fmol/mg of protein; and *K*_d_ = 21.0 nM and B_max_ = 656 fmol/mg of protein, respectively). In addition, saturation of the SK-N-SH protein nuclear fraction with [^3^H]-DTG was consistent with data from the saturation analysis of the proteins eluted from the affinity chromatography column. This result further supported the association of PB28 with histone proteins, which are mostly expressed in the nucleosome core particle within the nucleus (*K*_d_ = 18.2 nM; B_max_ = 830 fmol/mg of protein). Nonetheless, it was unclear if in the affinity chromatography process, the immobilized PB28 bound individual histone monomers, oligomers, or denatured forms of the nucleosome. Therefore, homology models of individual histones, or histone dimers, tetramers, and the nucleosomal core particle, were built in order to rationalize PB28 binding to these proteins (Abate et al., 2010). While a few docking poses were detected for PB28 at the H2A, H2B, and H3.3a histone monomers; the H3.3a/H4 dimer; and the core octamer, the most plausible interactions were observed with the H2A/H2B dimer. Consistently with the experimental data from the affinity chromatography experiment, no significant interactions were predicted between PB28 and the H4 monomer. Two sites on the H2A/H2B dimer were observed for preferential binding of PB28. Interestingly, these sites have roles in compacting chromatin fibers, through modulation of interactions between H2A/H2B and the H3.3a/H4 tetramer during nucleosomal assembly. In order to ascertain the ability of PB28 to access the nucleus and interact with the histone dimer, intracellular accumulation of the previously synthesized [^3^H]-PB28 (Colabufo et al., 2008a) was studied in SK-N-SH neuroblastoma and MCF7dx breast tumor cells, while PB28 binding with the reconstituted H2A/H2B dimer was verified. PB28 cell accumulation was up to fivefold higher in nuclear fractions than in the cytoplasm, while competitive binding of PB28 versus [^3^H]-PB28 at the H2A/H2B dimer resulted in a subnanomolar affinity value (IC_50_ = 0.50 nM), despite a high nonspecific binding (Abate et al., 2010). Therefore, PB28 was shown to be able to enter the nucleus and bind to the H2A/H2B histone dimer, suggesting that this molecule may act (also) through the modulation of nucleosomal assembly and/or chromatin compaction, likely influencing gene expression.

### Structure–Affinity Relationship (SAfiR) Studies

Based on the PB28 structure, a number of structure–affinity relationship studies were undertaken with the synthesis of diverse classes of ligands. The cyclohexyl group at the piperazine nucleus was removed or replaced with other alkyl or aryl substituents. Removal of the cyclohexyl ring (Berardi et al., 2009b) as well as change with aryl rings (Perrone et al., 2000) led to a 1000-fold drop in the affinity at both the receptor subtypes (sigma-1 *K*_i_ = 218 nM; sigma-2 *K*_i_ = 590 nM), with the worst affinity recorded for the 1-naphthalenyl derivative at the sigma-1 receptor (*K*_i_ = 2,480 nM). A 10- to 50-fold reduction in the affinity was also recorded for the alkyl derivatives (methyl, *n*-propyl, *i*-pentyl, and benzyl bearing compounds) at the sigma-1 receptor, whereas the drop was more drastic for the sigma-2 receptor with the methyl- and benzyl-containing analogs displaying the highest *K*_i_ values (*K*_i_ = 663 nM; *K*_i_ = 1870 nM).

**FIGURE 2:**
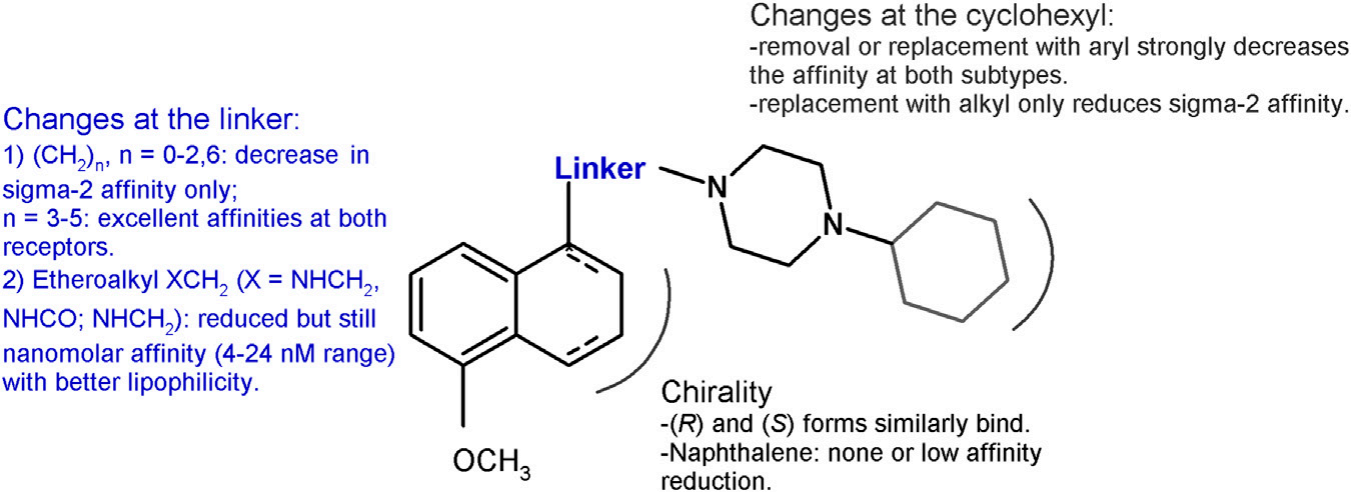
Graphic recapitulation of SAfiR studies involving the linker, the chirality and the cyclohexyl portions of PB28.

The propyl chain connecting the tetralin portion with the cyclohexylpiperazine was shortened or elongated (from 1 to 6 methylenes) or completely removed (Berardi et al., 2004). Shorter chain derivatives were characterized by a decreased affinity only for the sigma-2 subtype (*K*_i_ values ranging from 7.9 to 21.1 nM). By contrast, in the longer chain derivatives, an important drop in the affinity was recorded only for the hexamethylene-bearing compound at the sigma-2 receptor (*K*_i_ = 103 nM), while a low nanomolar or subnanomolar affinity was kept at the sigma-1 subtype. In particular, the compound bearing a 5-methylenes chain displayed affinity values comparable to PB28 at both subtypes. The important lipophilicity of PB28 (logP = 5.08) can be one of the causes of the high nonspecific binding in the brain of ^11^C-PB28 radiotracer (Kassiou et al., 2005). Thus, with the aim of reducing PB28 lipophilicity and in order to meet the requirements for the development of PET radiotracers for tumor imaging (Mach et al., 2013), an amine, amide, or ether function was alternatively inserted in the propylene chain at the 1-position on the tetralin (Abate et al., 2011d). Compounds binding both sigma receptor subtypes with appreciable nanomolar affinities (*K*_i_s ranging from 3.90 to 23.9 nM) and more appropriate lipophilic values were obtained, despite a 10- to 60- fold reduction of the affinity compared to PB28. Because PB28 was first synthesized as a racemic mixture, the two enantiomers were developed showing no enantioselectivity for both sigma receptor subtypes (Abate et al., 2011d). The lack of a preference by the sigma receptors for one of the two enantiomers at the 1-position of the tetralin was also confirmed by results from shorter-chain derivatives (Abate et al., 2009), and from the PB28 derivatives bearing the etheroatoms (amine, amide, ether) in the linker. For the latter compounds, only modest preferences (two- to fourfold) could be observed for one versus the other enantiomer (Abate et al., 2011d). The chirality was removed by oxidation of the tetralin nucleus to naphthalene, and, again, sigma high affinity ligands were obtained, but with an about three- and tenfold reduced affinity for the sigma-1 and sigma-2 receptor, respectively, compared to PB28 (Berardi et al., 2004). The same change (naphthalene replacing the tetralin nucleus) in the etheroalkyl-PB28 derivatives maintained similar affinity values as in their tetralin counterpart (Abate et al., 2011d). These changes and the corresponding results are summarized in Figure 2.

The methoxy group was unimportant in terms of sigma receptor affinity, as either on the tetralin or on the naphthalene nucleus the absence of the methoxy group was not detrimental for both subtypes (Berardi et al., 2004). Noteworthy, the unsubstituted tetralin and naphthalene bearing a 4-methylenes chain were characterized by improved subnanomolar affinity for the sigma-1 receptor and reduced affinity for the sigma-2, with the tetralin derivative characterized by the highest sigma-1 receptor affinity (*K*_i_ = 0.035 nM) (Berardi et al., 2004). On the other hand, the position of the methoxy group was determined. The 6-methoxy group on the tetralin ring appeared to select the sigma-1 over the sigma-2 receptor (*K*_i_ = 0.36 and 5.42 nM, respectively), whereas the 7-OCH_3_ was particularly detrimental for the sigma-1 subtype (*K*_i_ = 9.04 nM). By contrast, the same changes in the naphthalene ring led to a subnanomolar sigma-1 affinity with the 7-methoxy substituent. On the basis of these results, a diverse series of sigma-1 selective ligands were developed as 6-methoxy-tetralin and 6-methoxy-naphthalene derivatives (Berardi et al., 2005; Ferorelli et al., 2007; Niso et al., 2013b; Niso et al., 2019). In addition, the methoxy group was replaced by a hydroxy one with a 10-fold reduction for the 5-OH derivatives and an unchanged subnanomolar affinity for the 6-OH–bearing compounds compared to PB28. The same changes in the position of the methoxy or hydroxy substituents in the naphthalene series led to a 10- to 40-fold reduction in the affinity at both sigma subtypes, with the only exception of the 7-OCH_3_-bearing naphthalene, that displayed a subnanomolar affinity at the sigma-1 receptor (*K*_i_ = 0.9 nM), in contrast to its tetralin counterpart.

**FIGURE 3:**
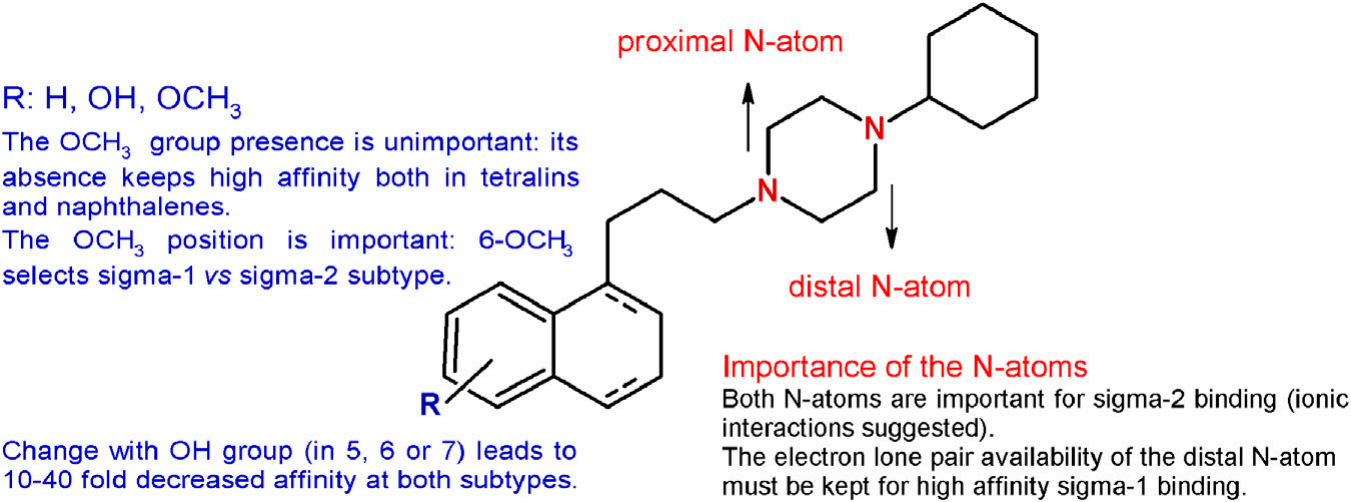
Graphic recapitulation of SAfiR studies involving the substituents on the tetralin/naphthalene bicycle and the N-atoms basicity of the piperazine ring.

Given the need of one basic nitrogen for the interaction with both receptor subtypes (Glennon, 2005), one of the two basic N-atoms (identified as proximal and distal in Figure 3) of the piperazine ring was alternatively replaced with a carbon atom, through the synthesis of the corresponding 1- and 4- cyclohexylpiperidine–bearing derivatives. The basicity was also reduced by incorporation of the N-atoms within an amide function through the synthesis of the PB28 propionamide and cyclohexylamide counterparts. The affinity for the sigma-2 receptor decreased for both the PB28 piperidine (from 50- to 100-fold) and amide derivatives (from 250- to 500-fold), suggesting that both N-atoms are important for high affinity binding with the sigma-2 subtype. Notably, the same is not true for the sigma-1 subtype. While the sigma-1 receptor binding drastically dropped for the 4-cyclohexylpiperidine and the 4-cyclohexanecarbonylpiperazine derivatives (*K*_i_s = 143 nM, and >5,000 nM, respectively), excellent sigma-1 affinity values (*K*_i_s = 1.1 and 0.11 nM, respectively) were obtained when the basicity of the distal N-atom was kept as in PB28 analogues bearing 1-cyclohexylpiperidine and 1-propionyl-4-cyclohexylpiperazine. In particular, the latter compound displayed an excellent profile as a sigma-1 selective ligand with its subnanomolar sigma-1 affinity and a 1600-fold selectivity against the sigma-2 subtype. The same conclusion could be drawn from the quaternarization of the piperazine N-atoms in the corresponding methylammonium salts: the “distal” N-atom lone pair must be available for interaction with the sigma-1 receptor, otherwise resulting in a decreased affinity (*K*_i_ = 338 nM). On the other hand, quaternarization of one of the N-atoms led to a just modest (10-fold) drop in the affinity for the sigma-2 subtype, supporting an ionic interaction of the ligands at the sigma-2 binding site (Berardi et al., 2009b). These structural changes and the corresponding results are summarized in Figure 3.

**FIGURE 4:**
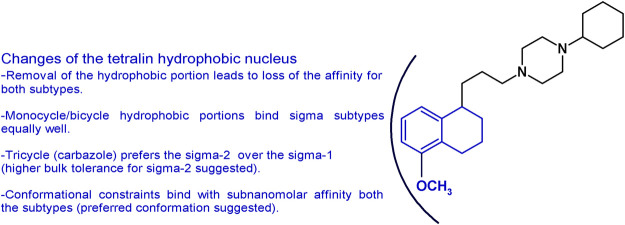
Graphic recapitulation of SAfiR studies involving the replacement of the tetralin/naphthalene hydrophobic nucleus.

The importance of the tetralin/naphthalene nucleus was also investigated by keeping the 1-propyl-4-cyclohexylpiperazine structure and removing the hydrophobic portion. In accordance to the pharmacophoric models of both receptor subtypes, the lack of the hydrophobic moiety led to the complete loss of the affinity (Glennon, 2005; Berardi et al., 2009b). Replacement with a bulky N-carbazolyl moiety led to a drastic drop in the sigma-1 affinity (*K*_i_ = 3,450 nM) but a notable sigma-2 receptor affinity (*K*_i_ = 12.6 nM). On the other hand, the less bulky N-indolyl (with one fused benzene ring less compared to the carbazole) had similar single-digit nanomolar affinity at both sigma subtypes (sigma-1 *K*_i_ = 3.28 nM; sigma-2 *K*_i_ = 1.90 nM). These data together suggest a less tolerant region for hydrophobic substituents in the sigma-1 compared to the sigma-2 subtype binding pocket (Mésangeau et al., 2011). Consistently, other hydrophobic portions obtained by insertion of one or two etheroatoms in the tetralin/naphthalene structure (such as chromane, isoquinolinone, or 1,8-naphthyridine rings) conferred just a low decrease in the affinity with low nanomolar affinity compounds at both subtypes (*K*_i_s from 2 to 26.6 nM ranges) (Abate et al., 2011a; Abate et al., 2011d; Abate et al., 2012). The data obtained gathering inspiration from siramesine, one of the sigma-2 reference compounds, were in line with the aforementioned results reported. The tetralin/naphthalene portion was replaced by the indole-3-yl portion or the N-(4-fluorophenyl)-indole-3-yl (*K*_i_s = from 6.50 to 28.9 nM) (Niso et al., 2013a). Replacement of the bicyclic with a monocyclic hydrophobic structure, such as a 2-amino-pyridine or a simple cyclohexyl or a substituted benzamide, kept the affinity at both receptors in the low nanomolar range, although a general reduction compared to PB28 was observed (*K*_i_s ranging from 0.25 to 45.5 nM) (Berardi et al., 2009b; Abate et al., 2011a; Abate et al., 2012). Of interest, the benzamide compounds were endowed with good sigma receptor affinity when an intramolecular H-bond could be formed between the amide-NH and OCH_3_ group on the benzene ring. This observation suggests that the benzamide series binds sigma receptors upon formation of a bicyclic structure. Isomers lacking the possibility of such intramolecular H-bond formation display at least a 10-fold reduced affinity (Abate et al., 2011a). PB28 constraints were also built by replacing the tetralin-1-yl-propyl portion with differently substituted 4-phenylcyclohexyl ones. In this series, exceptional mixed sigma receptor ligands were obtained. Several compounds displayed subnanomolar affinity for sigma-1 subtype (even 10-fold higher than PB28 affinity), and 1-digit nanomolar affinity for the sigma-2 receptors, providing hints about the preferred conformation for the interaction with the sigma receptor binding site. These structural changes and the corresponding results are summarized in Figure 4.

As for the changes on the cyclohexylpiperazine basic moiety with other moieties, the only result noteworthy was the affinity conferred by the 6,7-dimethoxytetrahydroisoquinoline. The presence of the latter is consistently strongly detrimental for the sigma-1 binding, whereas low nanomolar affinity values for the sigma-2 receptor are maintained when the basic moiety is linked to hydrophobic portions (Niso et al., 2013a; Abate et al., 2015b).

**FIGURE 5:**
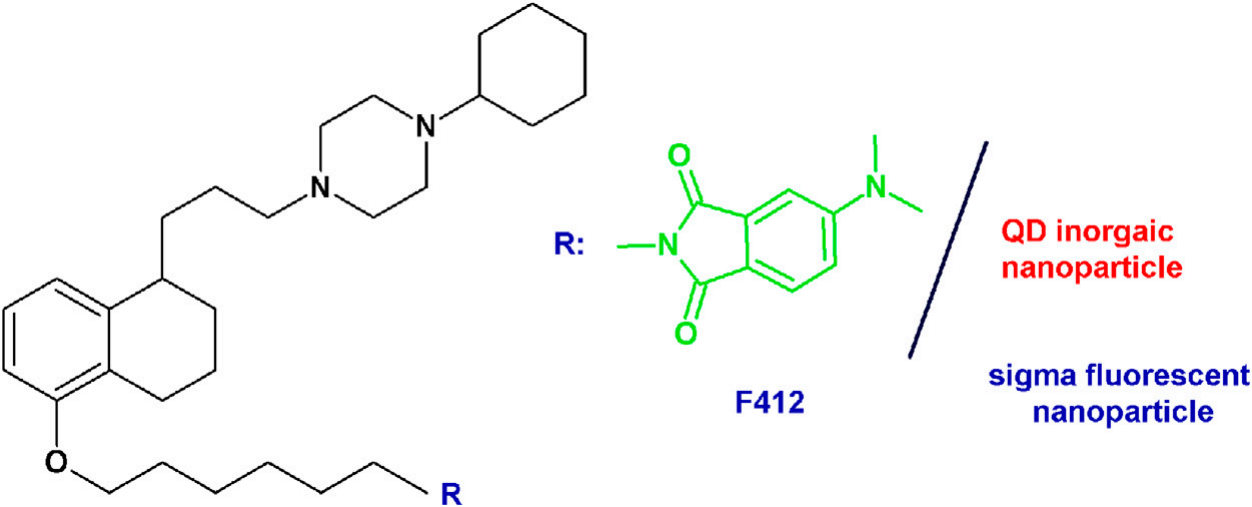
F412 small molecule and the corresponding schematization of the QD nanoparticle as sigma receptor fluorescent tracers.

*PB28-based fluorescent tracers*: Given the need of biological tools to study the “enigmatic” sigma receptors, the PB28 structure was modified with the aim of obtaining fluorescent tracers. Previous structural changes allowed one to identify the position that could be modified without an important decrease in sigma receptor affinity (Abate et al., 2011b; Abate et al., 2014). The highest affinity fluorescent ligand was obtained when the methoxy group on the tetralin was replaced by a hexamethylenoxy linker carrying in the ω-position the 4-(*N*,*N*-dimethylamino)phthalimide (F412; sigma-1 *K*_i_ = 5.37 nM; sigma-2 *K*_i_ = 6.9 nM) (Figure 5). This green emitter compound was successfully used in flow cytometry and confocal microscopy analyses to visualize sigma-2 receptors (Abate et al., 2014; Niso et al., 2015; Pati et al., 2017a; Pati et al., 2018a). Importantly, using this same strategy, also sigma-2 versus sigma-1 (and vice versa), selective ligands were obtained starting from the diverse pharmacophores identified through the years, such that fluorescent tracers to selectively study one sigma subtype at a time are also available (Niso et al., 2015; Abate et al., 2016; Liu et al., 2019; Cantonero et al., 2020). In order to expand the fluorescent properties, the same scaffold adopted for F412 was used to decorate inorganic quantum dots (QDs) generating nanoparticles with superior fluorescent properties able to visualize sigma receptors (Figure 5) (Pati et al., 2018a).

According to a multitarget ligands approach, the PB28 structure has also been modified in order to incorporate metal chelating portions and/or P-gp interacting moieties in order to increase the antitumor potentials with encouraging results, in particular from pancreatic tumors (Niso et al., 2013a; Pati et al., 2015; Pati et al., 2018b).

## Conclusion

We have herein presented the results obtained during the years with our lead compound and reference sigma receptor modulator PB28. Since PB28 binds equally well the sigma receptor subtypes (with subnanomolar affinity), it is important to elucidate whether its anti–SARS-CoV-2 activity is due to the interaction with one of the two subtypes, or both, and what interaction type is needed (agonist versus antagonist), although an off-target effect cannot be ruled out. Herein, we share our knowledge about PB28 pharmacology and provide structural elements that may help to drive future modifications on PB28 structure to optimize it toward a therapeutic drug. Additionally, we provide information about the structure–affinity relationship studies that have led to the generation of several PB28 derivatives, some of which display high nanomolar affinity and selectivity toward one of the two subtypes. The availability of these small molecules, together with PB28-based fluorescent tracers may be of further help to the scientific community working against the COVID-19 pandemic to better define the sigma receptor subtypes involvement in the antiviral action and quickly move forward. It is worth noting that the chaperone nature of the sigma-1 receptor (and likely of the sigma-2 receptor, as well) often makes the activity of the sigma receptor ligands cell-dependent. Therefore, we can expect that the affinity values of sigma receptor ligands do not necessarily reflect the antiviral activity. Therefore, structure antiviral-activity studies with the molecules herein presented and other molecules will be of extreme aid to formulate hypotheses about the sigma receptor involvement in the action against SARS-CoV-2. The results will possibly shed light on the mechanisms of interaction that do not only involve sigma receptor affinity, but also take into account the possible sigma receptor client proteins.

## Author Contributions

CA designed and drafted this work, NC and FB critically revised it for intellectual content, MN and MC contributed to conception and analyzed and interpreted the biological sections, and FA contributed to collection of data and revisions.

## Conflict of Interest

The authors declare that the research was conducted in the absence of any commercial or financial relationships that could be construed as a potential conflict of interest.
